# The use of fosmid metagenomic libraries in preliminary screening for various biological activities

**DOI:** 10.1186/s12934-014-0105-4

**Published:** 2014-07-22

**Authors:** Agnieszka Felczykowska, Aleksandra Dydecka, Małgorzata Bohdanowicz, Tomasz Gąsior, Marek Soboň, Justyna Kobos, Sylwia Bloch, Bożena Nejman-Faleńczyk, Grzegorz Węgrzyn

**Affiliations:** 1 Department of Molecular Biology, University of Gdańsk, Wita Stwosza 59, Gdańsk, 80-308, Poland; 2 Faculty of Biotechnology and Food Sciences, Slovak University of Agriculture in Nitra, Tr. A. Hlinku 2, Nitra, 949 76, Slovakia; 3 Institute of Oceanography, University of Gdansk, Al. Marszałka Piłsudskiego 46, Gdynia, 81-378, Poland

**Keywords:** Metagenomic libraries, Cyanobacteria, Escherichia coli cell factories, Bioactive compounds

## Abstract

**Background:**

It is generally believed that there are many natural sources of as yet unknown bioactive compounds with a high biotechnological potential. However, the common method based on the use of cell extracts in the preliminary screening for particular molecules or activities is problematic as amounts of obtained compounds may be low, and such experiments are hardly reproducible. Therefore, the aim of this work was to test whether a novel strategy to search for previously unknown biological activities can be efficient. This strategy is based on construction of metagenomic libraries and employment of *Escherichia coli* strains as cell factories producing compounds of properties potentially useful in biotechnology.

**Results:**

Three cyanobacterial metagenomic libraries were constructed in the fosmid system. The libraries were screened for various biological activities. Extracts from selected *E. coli* clones bearing constructs with fragments of cyanobacterial genomes revealed antimicrobial or anticancer activities. Interestingly, stimulation of growth of host bacteria bearing particular plasmids with certain cyanobacterial genes was detected, suggesting a potential possibility for improvement of *E. coli* cultivation during biotechnological production. The most interesting plasmids were sequenced, and putative mechanisms of biological effects caused by cyanobacterial gene products are discussed.

**Conclusions:**

The strategy of exploring cyanobacteria as sources of bioactive compounds, based on *E. coli* cell factories producing compounds due to expression of genes from metagenomic libraries, appears to be effective.

## Background

It is generally believed that organisms, mainly algae and higher plants, living in their natural habitats, are sources of many unknown compounds which can be of high biotechnological and pharmacological potential. However, commonly used methods of preliminary screening for interesting biological activities in tested organisms, based on analysis of cell or tissue extracts, are problematic due to their limited efficiency and reproducibility (see next paragraphs for details).

Cyanobacteria, called also blue-green algae, appear to be potentially rich sources of bioactive compounds [[1]]. They are photosynthesizing prokaryotic organisms, occupying marine, freshwater and terrestrial habitats, and belong to the oldest biological groups on Earth. It appears that marine cyanobacteria may be particularly rich in biotechnologically and pharmacologically useful molecules [[2]], however, it is considered that they are underexploited in this matter [[3]]. On the other hand, in recent years, an interest in bioactive compounds derived from these microorganisms increased significantly [[4]]. This is perhaps, at least in part, due to publication of papers reporting isolation of various compounds from cyanobacteria, including those possessing antiviral, antibacterial, antifungal and anticancer activities (summarized in ref. [[5]]).

Exploration of cyanobacterial sources is, however, neither easy nor simple. Despite evident successes in the use of these organisms in production of novel bio-fuels [[6],[7]], previous studies on identification of bioactive compounds in blue-green algae concentrated mostly on characterization of their cellular extracts. Such a strategy was effective in detection of secondary metabolites with interesting properties [[8],[9]]. However, the use of extracts has an important drawback, namely, production of certain compounds may depend on specific physiological conditions, thus, it may be difficult to repeat the exact conditions under which particular compounds were found in extracts. Moreover, efficiency of extraction of particular compounds from samples withdrawn from a natural habitat may be of low efficiency, and such experiments can be hardly reproducible due to changing environmental conditions. Therefore, a need for construction of cyanobacterial metagenomic libraries to discover previously unknown functional genes involved in biosynthesis of biotechnologically relevant compounds has been postulated [[5]]. Nevertheless, despite determination of full nucleotide sequences of genomes of many cynaobacterial strains [[10]], the use of genomic and metagenomic approaches in searching for bioactive compounds derived from cyanobacteria was not extensive. In fact, most such methods were employed to detect genes coding for enzymes revealing particular, desired features [[11],[12]], rather than to detect novel biological activities.

The aim of this work was to test whether a novel strategy, based on the use fosmid metagenomic libraries to search for biological activities appearing in *Escherichia coli* host cells bearing plasmids with fragments of cyanobacterial genomes, can be effective in detecting biotechnologically interesting features. If yes, this strategy might be used in subsequent works for isolation and characterization of compounds revealing particular activities, which would be significantly facilitated by availability of specific clones, eliminating potential problems with the amount of tested compounds and reproducibility of results. Efficient construction of the libraries was possible, between others, due to employment of a recently optimized method for isolation and purification of genomic DNA from filamentous cyanobacteria, suitable for construction of genomic libraries [[13]]. Such procedures are challenging due to production by cyanobacteria of large amounts of cellulose, pectins, murein and xylose, which are components of the cell wall. Moreover, these microorganisms synthesize and excrete complex polysaccharides and proteins which form mucous envelope and the protein S layer [[14],[15]]. All these compounds interfere with commonly used procedures of DNA isolation and purification. The improved method allowed us to overcome most of these problems [[13]] which facilitated efficient construction of cyanobacterial metagenomic libraries and their use in searching for biological activities.

## Results and discussion

### Construction of cyanobacterial metagenomic libraries

Three fosmid-based libraries of cyanobacterial metagenomes have been constructed. They were prepared using genomic DNA, isolated according to previously published procedure [[13]], derived from: (a) a bloom of *Nodularia* sp. which occurred in Gdańsk Bay, Baltic Sea (Poland) in June 2009; library 1; (b) a bloom of *Nodularia* sp. and *Aphanizomenon flos-aquae* which occurred in Gdańsk Bay, Baltic Sea (Poland) in May 2011; library 2; and (c) cultures of 5 marine cyanobacteria, isolated from Baltic Sea (deposited in Culture Collection of Northern Poland, CCNP, at University of Gdańsk, Poland) and cultured in laboratory: *Microcystis aeruginosa* CCNP 1101, *Microcystis aeruginosa* CCNP 1102, *Microcystis* aeruginosa CCNP 1103, *Anabaena sp*. CCNP 1406, *Synechocystis salina* CCNP 1104; library 3. Characteristics of these libraries are provided in Table 1. Considering an average sizes of cyanobacterial genome (reported to be between 1.44 and 9.05 Mb [[10]]), it was calculated that about 600 clones in the fosmid-based library (with average size of the insert of 40 kb) should cover efficiently a whole single genome. The obtained clones in each library were, therefore, sufficient to cover at least several cyanobacterial genomes.

**Table 1:** Cyanobacterial metagenomic libraries constructed in this work

**Library**	**Source of DNA**	**Number of obtained clones**^**a**^
Library 1	Bloom of *Nodularia* sp.; Gdańsk Bay, Baltic Sea (Poland), June 2009	1,806
Library 2	Bloom of *Nodularia* sp. and *Aphanizomenon flos-aquae*; Gdańsk Bay, Baltic Sea (Poland), May 2011	2,234
Library 3	Mixed cultures of: *Microcystis aeruginosa* CCNP 1101, *Microcystis aeruginosa* CCNP 1102, *Microcystis* aeruginosa CCNP 1103, *Anabaena sp*. CCNP 1406, *Synechocystis salina* CCNP 1104	~30,000

^a^Number of clones appearing after infection of *E. coli* host cells with 1/10 of volume of fosmid lysate obtained during the library construction.

As indicated above, two libraries (no. 1 and 2) were constructed with the use of DNA isolated from environmental samples of biological material, whereas library 3 contained DNA isolated from a mixture of 5 strains (to increase variability of the library) of cyanobacteria cultured in laboratory after their isolation from a natural habitat. As shown in Table 1, cultivation of cyanobacteria under laboratory conditions enhanced efficiency of metagenomic library construction (~30.000 clones vs. ~2.000 clones). We suggest that this might arise from contaminations of the environmental samples, which could interfere with efficiency in DNA isolation and purification, and/or cloning procedures.

### Inhibition of bacterial growth by extracts from library clones

In preliminary tests, extracts from 200 clones, randomly taken from constructed libraries, were prepared. These extracts were assessed for their effects on growth of various bacteria (strains of *Serratia marcescens*, *Pseudomonas aeruginosa*, *Staphylococcus aureus*, *Micrococcus luteus*, *E.coli* K-12, and *E. coli* O157:H7 were employed) in 96-well plates. More detailed analyses were performed in liquid bacterial cultures. It was found that 1% extract from host bacteria bearing the clone 123–2 inhibited growth of *S. marcescens* and *E.coli* B by 45% and 40%, respectively, as assessed by measurement of optical density of bacterial cultures. Although this inhibition of growth was not dramatic, it is important to note that concentration of the extract was relatively low (1%). Thus, it is likely that identification of the inhibitory agent should allow to clone appropriate gene(s) in an expression vector, its production in large amounts, and its potential use to achieve significant inhibition of bacterial growth.

### Stimulation of host bacterium growth by the presence of specific clones from the libraries

Although for medical application it is useful to identify antibacterial activities, for biotechnological purposes, it is often desirable to stimulate growth of bacteria which are employed to express recombinant genes and synthesize particular products. Therefore, we have also tested effects of the presence of clones from the libraries on growth of host *E. coli* cells. Since it is known that the presence of plasmids, especially relatively large constructs, may influence bacterial metabolism, physiology and growth [[16],[17]], in control experiments, a strain bearing a plasmid analogous to those containing fragments of cyanobacterial genomes, but carrying a fragment of *E. coli* genome (the 105–2 clone), was used.

**Figure 1:**
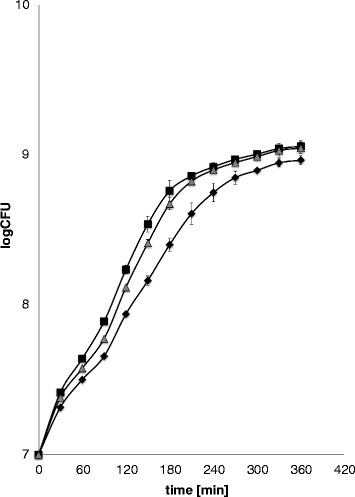
**Growth of*E. coli*cells bearing different clones from libraries.** The growth (37°C, LB medium) of bacteria bearing a control clone 105–2 (diamonds), or clone 123–3 (squares) or 129–3 (triangles) from the cyanobacterial metagenomic library was monitored. Mean values from 3 independent experiments are shown with error bars indicating SD. Calculated generation times at the mid-exponential phase of growth were 36 min for bacteria bearing 105–2, 28 min for bacteria bearing 123–3, and 28 min for bacterial bearing 129–3.

Perhaps surprisingly, it was found that in the presence of clone 123–3 or 129–3, the growth of *E. coli* host was stimulated rather than inhibited (Figure 1). Generation time at mid-exponential phase of growth was 36 min for the control strain, while it was calculated as 28 min for cells bearing either 123–3 or 129–3 clone. This indicates that expression of some cyanobacterial genes may enhance growth of *E. coli* cells which may be potentially useful in construction of new host strains to be used as microbial cell factories for biotechnological production of certain compounds.

### Anticancer activity of extracts from libraries’ clones

**Figure 2:**
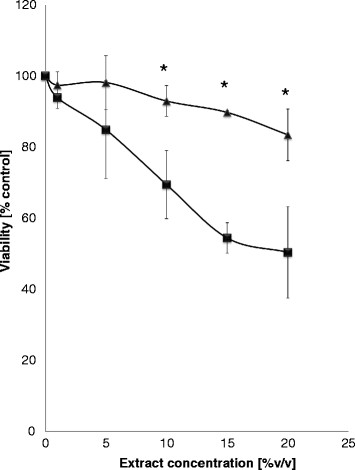
**Effects of extracts from library clones on viability of human cells.** Viability of wild-type HDFa (triangles) and cancer MCF-7 (squares) cells was assessed in media supplemented with various amounts of the extract from bacteria bearing the 123–3 clone. Mean values from 3 independent experiments are shown with error bars indicating SD. Statistically significant differences (p < 0.05 in the t-test) between two cell lines are indicated by asterisks. Viability of MCF-7 cells in the presence of the extract was significantly (p < 0.05) decreased relative to cultures without the extract at the same concentrations, thus no additional symbols are shown. The presence of the extract did not cause any significant decrease in viability of HDFa cells at all tested concentrations (p > 0.05).

To test anticancer activity, effects of 370 clone extracts, prepared as described above (for testing inhibition of bacterial growth), on viability of cancer cells (the MCF-7 cells line) and normal human fibroblast cells (the HDFa cell line), were assessed. After preliminary screening, and following more detailed analyses, it was found that extract from clone 123–3 significantly decreased viability of cancer cells in a dose–response manner (Figure 2). Importantly, effects of this extract on normal human cells were negligible (Figure 2). These results suggest that expression of some cyanobacterial genes in *E. coli* causes production of anticancer compound(s), while having no significant effects on viability of normal human cells.

### Analysis of sequences of selected clones

Since some clones from cyanobacterial metagenomic libraries revealed interesting properties, nucleotide sequences of inserts of selected constructs were determined. Following clones were chosen: 123–2 (extract from bacteria bearing this clone caused inhibition of growth of *S. marcescens* and *E.coli* B), 123–3 (extract from bacteria bearing this clone caused decreased viability of cancer cells, but not normal human cells, and growth of bacteria bearing this clone was stimulated), and 129–3 (growth of bacteria bearing this clone was stimulated).

Nucleotide sequences of the selected clones were determined, deposited in GenBank (accession numbers: KJ769136, KJ769135, KJ769134 for 123–2, 123–3, 129–3, respectively), and analysis of open reading frames included in the fragments of cyanobacterial genomes was performed. Results of analyses of selected genes, discussed below, are presented in Tables 2, 3 and 4 (for 123–2, 123–3, and 129–3, respectively).

**Table 2:** Results of analysis of DNA sequence of the insert in clone 123–2

**ORF position in the clone**	**Putative gene**	**Significant similarity (%) to known gene from indicated organism**	**Description**
1381-497	*wcaG*	Not found	COG0451 Nucleoside-diphosphate-sugar epimerases [cell envelope biogenesis, outer membrane/carbohydrate transport and metabolism]
4578-5294	*tolQ*	75% *Sinorhizobium fredii* NGR234	COG0811 Biopolymer transport proteins [intracellular trafficking and secretion]
5304-5756	*tolR*	75% *Sinorhizobium fredii* NGR234	TIGR02801 biopolymer transport protein, TolR
8452-8955	*pasL*	74% *Sinorhizobium meliloti* 2011	PRK10802 Peptidoglycan-associated lipoprotein
15265-16134	*opaP*	67% *Sinorhizobium meliloti* 2011	COG3637 Opacity protein and related surface antigens [cell envelope biogenesis, outer membrane]
24299-23364	*rhaT*	67% *Sinorhizobium fredii* NGR234	COG0697 Permeases of the drug/metabolite transporter (DMT) superfamily [carbohydrate transport and metabolism/amino acid transport and metabolism]

Selected genes located in this DNA fragment are described.

**Table 3:** Results of analysis of DNA sequence of the insert in clone 123–3

**ORF position in the clone**	**Putative gene**	**Significant similarity (%) to known gene from indicated organism**	**Description**
401-1672	*gckA*	Not found	COG2379 Putative glycerate kinase [carbohydrate transport and metabolism]
2611-1691	*phpP*	Not found	PLN02645 phosphoglycolate phosphatase
4201-2720	*gatA*	76% *Rhizobium* sp. strain NT-26	COG0154 Asp-tRNAAsn/Glu-tRNAGln amidotransferase A subunit and related amidases [translation, ribosomal structure and biogenesis]
4976-4689	*gatC*	76% *Rhizobium* sp. strain NT-26	COG0721 Asp-tRNAAsn/Glu-tRNAGln amidotransferase C subunit [Translation, ribosomal structure and biogenesis]
5828-5118	*medH*	75% *Agrobacterium vitis* S4	PRK00685 metal-dependent hydrolase; Provisional
11125-12420	*pyrC*	67% *Rhizobium* sp. strain NT-26	cd01317 Dihydroorotase (DHOase), subgroup IIa; DHOases catalyze the reversible interconversion of carbamoyl aspartate to dihydroorotate, a key reaction in pyrimidine biosynthesis
20911-22095	*matA*	71% *Rhizobium leguminosarum* bv. trifolii CB782	Multidrug ABC transporter ATPase/protein_id = “AHG44931.1
22188-22355	*rpmG*	71% *Rhizobium* sp. str. NT-26	PRK00595 50S ribosomal subunit protein L33

Selected genes located in this DNA fragment are described.

**Table 4:** Results of analysis of DNA sequence of the insert in clone 129–3

**ORF position in the clone**	**Putative gene**	**Significant similarity (%) to known gene from indicated organism**	**Description**
1885-3162	*thrC*	73% *Starkeya novella* DSM 506	COG0498 Threonine synthase [amino acid transport and metabolism]
4778-5017	*rimL*	70% *Polymorphum gilvum* SL003B-26A1	COG1670 Acetyltransferases, including N-acetylases of ribosomal proteins [Ttanslation, ribosomal structure and biogenesis]
10222-8198	*phaS*	74% *Polymorphum gilvum* SL003B-26A1	TIGR01838 poly (R)-hydroxyalkanoic acid synthase, class I [fatty acid and phospholipid metabolism, Biosynthesis]
11838-13154	*homD*	69% *Polymorphum gilvum* SL003B-26A1	PRK06349 Homoserine dehydrogenase [synthesis of Met, Thr, Ile from Asp]
19761-21452	*arnT*	78% *Starkeya novella* DSM 506	COG1807 4-amino-4-deoxy-L-arabinose transferase and related glycosyltransferases of PMT family [amino acids and vitamins transport]
22371-22703	*lpxB*	76% *Mesorhizobium loti* MAFF303099	PRK01021 lipid-A-disaccharide synthase
23930-22725	*metC*	65% *Starkeya novella* DSM 506	PRK05967 cystathionine beta-lyase [methionine and cysteine metabolism]
24090-25163	*hisJ*	67% *Starkeya novella* DSM 506	COG0834 ABC-type amino acid transport/signal transduction systems, periplasmic component/domain [amino acid transport and metabolism/signal transduction mechanisms]
29290-27989	*cfaS*	70% *Mesorhizobium opportunistum* WSM2075	COG2230 Cyclopropane fatty acid synthase and related methyltransferases [cell envelope biogenesis, outer membrane]

Selected genes located in this DNA fragment are described.

The fragment included in the 123–2 clone contains several ORFs coding for putative enzymes (Table 2). Although no obvious candidate for a protein which, when liberated from cells, might inhibit bacterial growth was found, some enzymes might influence transport of particular compounds (putative products of *wcaG, tolQ, tolR, rhaT* genes), peptidoglycan structure (putative product of the *pasL* gene) or outer membrane function (putative product of the *opaP* gene). On the other hand, it might be not an enzyme but a product of its reaction that could negatively affect *S. marcescens* and *E.coli* B growth when present in cell extract. Definitely, in order to indentify compound(s) responsible for bacterial growth inhibition, it would be necessary to clone smaller fragments of the 123–2 construct, determine gene(s) responsible for specific effects, and investigate the mechanism of inhibition. This, however, was not the aim of this study which focused on testing whether cyanobacterial metagenomic libraries can be successfully employed in searching for various biological activities.

The clone 123–3 (Table 3) appeared the most interesting one among all tested in this study. First, extract from bacteria bearing this clone caused decreased viability of cancer cells, while having little effect on normal human cells. Second, growth of bacteria bearing this clone was stimulated. The latter effect might be potentially caused by activities of putative proteins encoded in the tested genome fragment, and involved in biosynthesis of proteins (*gatA*, *gatC*, *rpmG* ) and nucleotides (*pyrC*). Moreover, the transport of amino acids and carbohydrates from the medium could be intensified by *metA*, and *gckA* genes’ products. Stimulation of protein and nucleic acid syntheses might likely enhance growth rate of host bacteria. Nevertheless, actual mechanism of this phenomenon remains to be elucidated. Even more difficult is to predict the cause of anticancer action of extract from bacteria bearing the 123–3 clone. Although it seems more likely that product(s) of reaction(s) of enzyme(s) encoded in the insert of this clone may affect viability of cancer, but not normal, cells, one cannot exclude a possibility that some enzymes (like products of *phpP* and/or *medH* genes) themselves might interfere with functions of cells of the MCF-7 line.

Although stimulatory effects of the presence of clones 123–3 and 129–3 on growth of host cells were similar, two analyzed DNA fragments contain different ORFs. Nevertheless, DNA of 129–3 codes for several putative proteins involved in pathways of biosyntheses of proteins and lipids (*thrC*, *rimL*, *phaS, lpxB, cfaS, homD, metC*) and theirs transport (*arnT*, *hisJ*). Therefore, one may speculate that stimulation of anabolic processes might positively affect growth rate of *E. coli* host cells, which can have a potential impact on biotechnological production.

## Conclusions

Construction of cyanobacterial metagenomic libraries, and expression of genes from particular clones in *E. coli*, resulted in detection of antibacterial, anticancer, and bacterial growth stimulatory activities in extracts of host cells, as well as in the host cells themselves. Identification and characterization of particular active compounds require further analyzes, and specific mechanisms leading to observed effects remain to be elucidated, but one should note that they were not aims of the current work. The presented results provide a proof of concept that the strategy of searching for various bioactive compounds in clones from fosmid metagenomic libraries may be effective and successful.

## Methods

### Microbial strains

Following cyanobacteria were employed: *Nodularia* sp. isolated from a bloom which occurred in Gdańsk Bay (Baltic Sea, Poland) in June 2009, *Nodularia* sp. and *Aphanizomenon flos-aquae* isolated from a bloom which occurred in Gdańsk Bay (Baltic Sea, Poland) in May 2011, and following 5 strains isolated from Baltic Sea and deposited in Culture Collection of Northern Poland, CCNP, at University of Gdańsk (Poland): *Microcystis aeruginosa* CCNP 1101, *Microcystis aeruginosa* CCNP 1102, *Microcystis aeruginosa* CCNP 1103, *Anabaena sp*. CCNP 1406, and *Synechocystis salina* CCNP 1104.

Following bacterial strains were employed for testing antibacterial activities of extracts from libraries’ clones: *Serratia marcescens*, *Pseudomonas aeruginosa*, *Staphylococcus aureus*, *Micrococcus luteus*, *Escherichia coli* B, *Escherichia coli* O157:H7. Strains *S. aureus* DMB-pR1-20 and *P. aeruginosa* DMB-pR1-23 were isolated in the Medical University Hospital in Gdańsk, and identified with the use of VITEK Legacy (bioMérieux). Strain *M. luteus* DMB-pR1-1, isolated from the human throat [[18]], and strain *S. marcescens* DMB-pR1-2 were kindly provided by Prof. T. Kaczorowski, Department of Microbiology, University of Gdansk; the identification of strain *S. marcescens* DMB-pR1-2 was confirmed by Elżbieta Mączak (University of Gdańsk). Clinical strain *E. coli* O157:H7 was obtained from the Cincinnati Children’s Hospital Medical Center [[19]].

### Human cell lines

Human Dermal Fibroblast adult line (HDFa; Cascade Biologics, Portland, OR, USA) was employed. The human breast adenocarcinoma (MCF-7) cell line was obtained from the Department of Microbiology, Tumor and Cell Biology, Karolinska Institute, Sweden. These cell lines were used in experiments testing effects of extracts from clones of libraries on viability of human cells.

### Construction of cyanobacterial metagenomic libraries

Fosmid metagenomic libraries were constructed with the use of the CopyControl Fosmid Library Production Kit with pCC2FOS Vector (purchased from Epicentre Biotechnologies), according to manufacturer’s instructions. Cyanobacterial genomic DNA was isolated and purified as described previously [[13]], and 20 μg of DNA from each isolation experiment were used for library construction. The final product of each construction procedure was 1 ml of phage lysate which was then used to infect *E. coli* T1^R^ host strain (provided in CopyControl Fosmid Library Production Kit). Number of obtained clones in each experiment is provided in Table 1. Routinely, 0.1 ml of the lysate was used, and the rest (0.9 ml) is being stored for potential further use. For control experiments, a clone bearing the 41,285 bp fragment of *E. coli* genome, encompassing the region between *abc2T* and *pdpN* genes, was constructed and named 105–2.

### Preparation of extracts from library clones

Bacterial (*E. coli*) clones from a gene library were cultured in 10 ml of LB medium (Sigma) supplemented with Fosmid Library AutoInduction Solution (Epicentre Biotechnologies; 1:500, v/v) at 37°C for 16 h at 150 rpm. Then, the cultures were centrifuged (4,000 rpm, 10 min), and the pellets were suspended in 2 ml of 0.01 M Tris–HCl buffer pH 7.0 (Sigma). The mixtures were sonicated (pulsar = 20% of maximal amplitude, 6 min) and filtered with the use of CA membrane filters (Corning).

### Assessment of antibacterial activities of extracts

Preliminary tests were performed in 96-well plates. Bacterial strains were precultured in LB medium (Sigma) at 37°C for 16 h at 150 rpm. Suspensions of cultures were prepared to obtain a final inoculum of 2 × 10^5^ cfu/ml in each assay. The cultures were incubated in 96-well plate in the presence of gene library clone extracts at concentrations between 0.5 and 25% (v/v) for 24 h. Then, optical density was measured at 570 nm. Data were obtained from at least three independent experiments.

After preliminary screening, more detailed assays were performed in flask cultures. Bacterial strains were precultured and inoculated as described above. 30 ml of cultures in LB medium were incubated at 37°C, at 150 rpm, in the presence of gene library clone extracts at final concentration 1% (v/v) for 24 h. Optical density was measured at 600 nm in time intervals for 24 h. Data were obtained from at least three independent experiments.

### Estimation of effects of library clones on host cell growth

*E. coli* T1^R^ cells bearing particular library clone were grown as described in the preceding section. Number of bacteria per ml of culture was determined at indicated times by measurement of A_600_. Generation time of each culture was calculated from results obtained during mid-exponential phase of growth.

### Determination of human cell viability

MCF-7 cells were maintained in RPMI 1640 (Sigma) medium supplemented with 10% (v/v) fetal bovine serum and antibiotics (Sigma). HDFa cells were maintained in DMEM medium (Sigma) supplemented with 10% (v/v) fetal bovine serum (Sigma), antibiotics and 4 mM L-glutamine (Sigma).

Human cell viability was determined by the MTT method [[20]]. Briefly, cells were seeded at density of 4 × 10^3^ per well of 96-well plate, and allowed to attach overnight. The medium was replaced with a fresh one supplemented with 1, 5, 10, 15, or 20% (v/v) of library clone extract, and the incubation was continued for 24 h. Then, 25 μl of MTT solution (4 mg/ml; Sigma) was added to each well. After 3 h incubation, the medium was removed, and 100 μl of DMSO per well was added. Absorbance was measured at 570 nm (with reference wavelength 660 nm). Data were obtained from at least three independent experiments.

### DNA sequence determination and analysis

DNA sequences of selected clones were determined by commercial sequencing (Genomed S.A.). Sequences were analyzed using the BLAST software. ORFs were compared and analyzed using Clone Manager 7 (Sci-Ed Software).

## Abbreviations

CA: Cellulose acetate

cfu: Colony forming units

DMSO: Dimethyl sulfoxide

DMEM: Dulbecco’s modified eagle’s medium

LB: Lysogeny broth (also called Luria-Bertani broth)

MTT: 3-(4,5-dimethylthiazol-2-yl)-2,5-diphenyltetrazolium bromide

ORF: Open reading frame

RPMI: Roswell park memorial institute medium

## Competing interests

The authors declare that they have no competing interests.

## Authors’ contributions

AF made the metagenomic libraries, designed the experiments of antibacterial and anticancer activity of genome library clone extracts and performed the experiments of antibacterial activity in flask cultures. AD and MS screened the gene libraries for antibacterial activity. MB screened the gene libraries for anticancer activity. TG analyzed the sequences of 123–2, 123–3 and 129–3 clones. JK isolated and cultured the cyanobacterial strains. SB and BN assisted in analyzing the results of experiments. GW provided the general concept of the study, supervised the work and drafted the manuscript. All authors read and approved the final manuscript.
